# The role of perceived social support, self-efficacy, and psychological skills in psychological resilience: a theoretical model test on academy football players

**DOI:** 10.3389/fpsyg.2025.1716489

**Published:** 2026-01-12

**Authors:** Anıl Siyahtaş, Cemal Güler, Ataman Tükenmez, Cemile Nihal Yurtseven, Özge Ercan, Suzan Dal, Ali Kaya

**Affiliations:** 1 Turkish Football Federation, Istanbul, Türkiye; 2 Faculty of Sports Sciences, Istanbul University-Cerrahpaşa, Istanbul, Türkiye

**Keywords:** academy football players, perceived social support, psychological resilience, psychological skills, self-efficacy

## Abstract

**Background:**

Academy football players are continuously challenged in terms of their psychological resilience due to the high pressure competitive environment and developmental demands. In this context, key factors influencing athletes’ psychological resilience include perceived social support, self-efficacy, and psychological skills. This study examined the effect of perceived social support on academy football players’ psychological resilience, and the mediating roles of self-efficacy and psychological skills.

**Methods:**

Data were collected through voluntary surveys administered to football players in the academies of professional football clubs in Istanbul between August and September 2025. The study sample consisted of 430 academy players (*n* = 430) with a mean age of 15.67 ± 0.96 years. The factor structure of the measurement instruments was examined using Confirmatory Factor Analysis (CFA), and the relationships among variables were tested through Structural Equation Modeling (SEM).

**Results:**

The findings indicated that: (1) perceived social support had no direct significant effect on psychological resilience (95% CI [−0.015, 0.237]); (2) perceived social support exerted a significant direct effect on self-efficacy (*β* = 0.475; 95% CI [0.343, 0.589]); and (3) perceived social support indirectly and significantly influenced psychological resilience through self-efficacy and psychological skills (*β* = 0.226; 95% CI [0.158, 0.316]). These results suggest that perceived social support does not enhance resilience directly, but rather operates through athletes’ self-efficacy beliefs and psychological skills.

**Conclusion:**

This highlights the importance of both environmental support and individual psychological resources during the transition to professionalism. Accordingly, coaches, mentors, and support staff in football academies are encouraged not only to provide social support but also to implement programs aimed at fostering athletes’ self-efficacy.

## 1 Introduction

The competitive nature of modern football imposes substantial physical and psychological demands on academy players striving to reach the professional level (Rye et al., 2022). These athletes must cope with heightened performance expectations, intensive training schedules, injury risks, and uncertainty about their future careers (Gledhill et al., 2017). This demanding developmental pathway underscores that sustainable success in sport depends not only on physical abilities but also on psychological resilience (Gucciardi et al., 2015). Psychological resilience is commonly defined as an individual’s capacity to adapt, recover, and sustain performance in the face of stress and pressure (Hartmann et al., 2022). In high pressure sports such as football, resilience is regarded as a critical psychological resource enabling athletes to manage stressors both on and off the field (Fletcher and Sarkar, 2012). Recent research further suggests that the development of psychological resilience is shaped not only by individual attributes but also by environmental and relational factors (Cusack et al., 2016; Liang and Cao, 2021). In this context, perceived social support (Mira et al., 2023), self-efficacy (Kim et al., 2023), and psychological skills (Golby and Wood, 2016) have been identified as particularly influential resources for athletes.

The theoretical framework of this study draws on Bandura’s (1986) Social Cognitive Theory, with particular emphasis on the principle of Triadic Reciprocal Causation. This perspective asserts that human functioning is shaped through an ongoing interplay between environmental conditions, cognitive processes, and behavioral tendencies. In line with this proposition, the present study positions perceived social support as the primary environmental influence that provides athletes with essential external resources when they encounter stress. Consistent with Social Cognitive Theory, such environmental factors are expected to shape athletes’ cognitive processes, which in this research are represented by self-efficacy. Self-efficacy, in turn, is thought to guide the deployment of psychological skills, including self-regulation and goal-setting, that support resilient coping. Viewing self-efficacy as a cognitive bridge through which environmental resources are transformed into concrete behavioral strategies offers a clear theoretical rationale for the proposed sequential relationship from social support to self-efficacy and subsequently to psychological skills.

Perceived social support can directly influence individuals’ self-perceptions and self-efficacy beliefs, which in turn enhance their psychological resilience by increasing their ability to cope with challenges (Chan, 2020; Yin et al., 2022). Social support encompasses the emotional, cognitive, and evaluative resources individuals obtain through their relationships with others, and it plays a particularly critical role in the developmental process of young athletes (Fletcher and Sarkar, 2012; Bruner et al., 2021). Self-efficacy refers to an individual’s belief in their ability to succeed in a given situation, and it is a key determinant of motivation, effort, and persistence in the face of adversity (Kenioua and El-Kadder, 2016). Psychological resilience, meanwhile, is commonly defined as the capacity to adapt and recover in the face of stress, pressure, and negative life events (Galli and Gonzalez, 2015). Within this framework, perceived social support, self-efficacy, and psychological skills are conceptualized as fundamental psychosocial components that shape athletes’ resilience. Accordingly, the present study examines the sequential mediating roles of self-efficacy and psychological skills to explain the indirect effects of perceived social support on psychological resilience. The primary contribution of this research is the empirical testing of a complex, chained mediation model, a mechanism that, despite being theoretically supported by SCT, has not yet been comprehensively tested in a single model within the context of academy football. The proposed model provides a comprehensive theoretical framework that elucidates how perceived social support contributes to resilience through self-efficacy and psychological skills, while simultaneously testing the core assumptions of Social Cognitive Theory (Bandura, 1986). By focusing on academy level football players, this research seeks to address a critical gap in the sports psychology literature by empirically testing a theoretical model that integrates perceived social support, self-efficacy, and psychological skills as predictors of resilience. Furthermore, the findings are expected to offer practical implications for coaches, mentors, and practitioners by advancing understanding of the mechanisms that strengthen the psychological resilience of athletes during key developmental stages, providing a data-driven path for targeted intervention strategies.

## 2 Literature review and research hypotheses

### 2.1 The effect of perceived social support on self-efficacy

Perceived social support refers to an individual’s subjective perception that they are cared for and valued by significant others, such as spouses, family members, and friends, and that they have a reliable social network to rely on in times of need (Zhou et al., 2022; Chen and Che, 2024). Previous research has demonstrated that individuals with higher levels of perceived social support experience lower stress (Glandorf et al., 2022) and greater life satisfaction (Çakmak Yıldızhan and Demir, 2024). In the context of sports, higher levels of social support have been shown to enhance athletes’ motivation (Wibowo et al., 2024) and to be associated with reduced depressive symptoms (Sullivan et al., 2020). Moreover, social support has been identified as a critical factor in fostering self-efficacy (Zhang et al., 2022). Within the framework of Bandura’s (1997) Social Cognitive Theory, self-efficacy is defined as an individual’s belief in their capacity to succeed in a given task or situation. This belief exerts a direct influence on cognition, affective states, motivation, and behavior, thereby shaping both psychological adjustment and performance. Bandura (1997) critically identified four key sources of self-efficacy. Social support is directly linked to the source of verbal persuasion, where supportive communication from significant others (coaches, family) strengthens an athlete’s belief that they possess the skills necessary to succeed. This theoretical link is empirically supported by studies such as Adler-Constantinescu et al. (2013), who found a significant relationship between perceived social support and self-efficacy in adolescents. Their findings emphasize that the emotional and informational reassurance provided by the social network, in particular, serves as a form of verbal persuasion, leading to a stronger sense of personal efficacy. Consequently, self-efficacy is not solely an internal evaluation but a dynamic construct that is shaped and reinforced by feedback from the social environment. In particular, the support athletes receive from their social environment plays a decisive role in developing and strengthening their perceived efficacy. Emotional and informational support provided by coaches, teammates, family members, and the broader social environment enhances athletes’ beliefs in their own capabilities (Wang et al., 2024). Based on these considerations, the following hypothesis was proposed:

*H1:* Perceived social support positively predicts self-efficacy.

### 2.2 The effect of perceived social support on psychological resilience

Perceived social support has been associated with increased self-confidence and reduced fatigue in athletes (Yao et al., 2025). Research in sports psychology suggests that perceived support is a critical factor influencing athletes’ psychological processes (Gabana et al., 2017). Accordingly, social support based interventions aim to help athletes recognize existing support systems and strengthen their perceptions of support. Perceived support is considered an essential resource for maintaining both the physical and psychological wellbeing of athletes (Katagami and Tsuchiya, 2017). Several studies have examined the effects of social support on athletes’ psychological health. For instance, Malinauskas and Malinauskiene (2018) found that perceived social support significantly influences psychological wellbeing. Similarly, social support resources are crucial for promoting the psychological health of disabled athletes (Mira et al., 2023). Strengthening athletes’ perceptions of social support has been identified as an important element in enhancing psychological wellbeing (Latif et al., 2024). Building upon the framework of Bandura’s Social Cognitive Theory, the relationship between social support and resilience is deeply rooted in the concept of agency and self-efficacy. Support, particularly in the form of verbal persuasion, fundamentally reinforces an athlete’s belief in their ability to overcome adversity. This enhanced, challenge-specific self-efficacy is the core cognitive mechanism that drives psychological resilience. Psychological resilience is defined as the capacity of athletes to adapt and recover in response to challenging life events, such as stress, pressure, injury, and failure (Blanco-García et al., 2021). It is also regarded as a critical psychological resource for maintaining performance and safeguarding mental health (Hartmann et al., 2022). When social networks provide emotional safety and informational resources, athletes gain the necessary conviction to engage in proactive and problem-focused coping behaviors, which are the behavioral manifestations of high resilience (Bandura, 1997). However, the true mechanism linking perceived social support to resilience may be indirect, particularly in developmental contexts like football academies. Resilience, as an internal, self-regulatory trait (Rutter, 1987), is heavily reliant on the athlete’s capacity for independent coping. In academy environments, the constant pressure to perform and the necessity for the athlete to internalize problem-solving skills suggest that social support may not directly build resilience; rather, its primary function is to indirectly strengthen the athlete’s internal resources (self-efficacy and psychological skills) which then lead to adaptation. This suggests that the direct effect of perceived social support on psychological resilience may be non-significant, as the effect is almost entirely carried through the sequential cognitive and behavioral steps. Given the limited research on the direct relationship between perceived social support and psychological resilience, and the theoretical ambiguity regarding the direct path in developing athletes, the present study aims to address this gap. Accordingly, the following hypothesis was proposed:

*H2:* Perceived social support positively predicts psychological resilience.

### 2.3 Chain mediation of self-efficacy and psychological skills

Psychological resilience is a multidimensional construct reflecting an individual’s capacity to adapt to stress, pressure, and adversity, shaped through the interaction of both internal and external resources (Rutter, 1987). Among athletes, perceived social support is considered a critical external resource that nurtures this capacity and serves as an important determinant in the development of psychological resilience (Kara and Ceyban, 2025). However, research indicates that the relationship between perceived social support and psychological resilience is not merely direct; it can be strengthened and guided through various intervening psychosocial variables (Malinauskas and Malinauskiene, 2018; Liu et al., 2023). Based on Bandura’s Social Cognitive Theory, this overall effect unfolds sequentially, with self-efficacy acting as the initial cognitive mediator. Perceived social support functions as a key external resource that strengthens self-efficacy (Shi et al., 2025). This is the critical link: according to Bandura (1997), self-efficacy serves as a primary motivational and regulatory determinant of behavior. Higher self-efficacy (belief in one’s ability) dictates whether an athlete chooses to apply their learned psychological skills, how much effort they expend, and how long they persevere when facing obstacles. Therefore, the enhanced belief in capability (self-efficacy) translates directly into the effective utilization of psychological skills (Kazemi and Kohandel, 2015), including concentration, stress coping, problem-solving, and motivation maintenance. In addition to self-efficacy, psychological skills play a critical role in this process. These skills encompass athletes’ abilities to cope with challenges, maintain performance, and adapt to changing conditions. As the ultimate behavioral output in the cognitive-behavioral chain, the consistent and effective application of these psychological skills enables the athlete to manage stressful environments, regulate negative affect, and overcome performance setbacks. This successful engagement in problem-focused coping, which is the definition of mastering adversity, is the very mechanism that builds and demonstrates robust Psychological Resilience (Bandura, 1997). Numerous studies have demon-strated that athletes with higher self-efficacy exhibit stronger psychological resilience (Foster and Chow, 2020). Moreover, perceived social support has been identified as an important facilitator in the development of these skills (Rees and Freeman, 2007). Based on this framework, the present study posits that the effect of perceived social support on psychological resilience occurs not only directly but also through a sequential chain mediated by self-efficacy and psychological skills. Incorporating these two mediators in the chain mediation model provides a more comprehensive explanation of how social support influences psychological resilience. Accordingly, the following hypothesis is proposed:

*H3:* Self-efficacy and psychological skills play a sequential mediating role in the effect of perceived social support on psychological resilience.

## 3 Materials and methods

### 3.1 Research model

**Figure 1:**
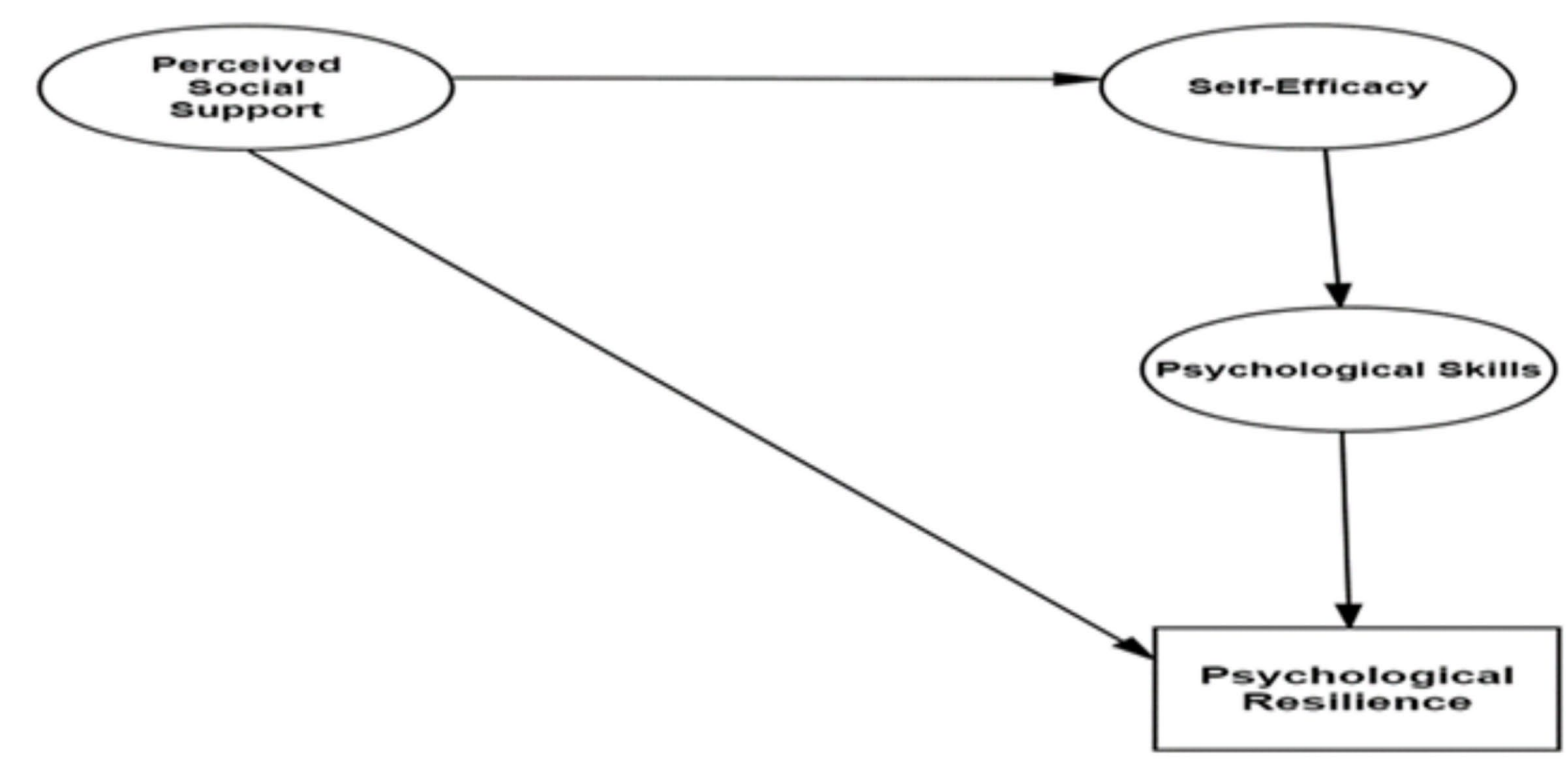
Research model.

This study examined the structural relationships among perceived social support, self-efficacy, psychological skills, and psychological resilience in academy football players using Structural Equation Modeling (SEM). In the proposed model, perceived social support was conceptualized as an independent latent variable, whereas self-efficacy, psychological skills, and psychological resilience were treated as dependent latent variable. A schematic representation of the hypothesized model is presented in Figure 1.

### 3.2 Research group

The study sample comprised 430 football players enrolled in the academies of professional football clubs in Istanbul, Türkiye (*n* = 430). Convenience sampling was employed for participant recruitment. The inclusion criteria were defined as follows: being a Turkish citizen, belonging to the academy squad of a professional club, holding an active athlete license, and voluntarily agreeing to participate in the study. To ensure the findings’ generalizability within the elite youth development context, further details regarding the competitive level are provided. In Türkiye, professional football consists of four competitive tiers: the Super League, 1st League, 2nd League, and 3rd League. The target population of the present research included academy players aged 15–19 competing within the youth structures of clubs from these professional leagues. The sample comprised players from clubs located in Istanbul. All participating athletes were actively competing in organized youth competitions, training at least twice per week, and playing one official match weekly, indicating a consistent engagement in structured, competitive football. Detailed information on the demographic characteristics of the academy football players is provided in Table 1.

**Table 1:** Demographic information.

Variable	*n*	%	*M* ± Ss
Educational status			
High school	416	96.7	
University	14	3.3	
Family income level			
Income less than expenses	24	5.6	
Income equals expenses	270	62.8	
Income exceeds expense	136	31.6	
Playing year			
1 year and under	7	1.6	
Between 2 and 4 years	156	36.3	
Between 5 and 7 years	188	43.7	
8 years and over	79	18.4	
Age			15.67 ± 0.96
Total	430	100	

Table 1 presents the demographic distribution of the academy football players participating in the study. The vast majority of the 430 football players participating in the study are receiving high school-level education (96.7%). Most participants belong to the middle-income group (62.8%), and the average age is 15.67 ± 0.96. When examining the distribution of licensed playing years, it is seen that a significant portion of the players (43.7%) have between 5 and 7 years of experience.

### 3.3 Data collection tools

Personal Information Form: This form has been prepared to obtain information about academy players’ age, education status, family income status, and the year they started playing licensed football.

Multidimensional Perceived Social Support Scale (MPSSS), a measurement tool consisting of a total of 12 items that allows individuals to subjectively assess the adequacy of the social support they perceive from three different sources: family (4 items), friends (4 items), and a special person (4 items). The scale was developed by Zimet et al. (1988). The adaptation to Turkish was carried out by Eker and Arkar (1995). The Turkish version of the scale was revised by Eker et al. (2001), and validity and reliability analyses were conducted on this version. Each item on the scale is rated using a 7-point Likert-type scale. High scores on the scale indicate a high level of perceived social support. As a result of the confirmatory factor analysis conducted in this study, the factor structure of the scale was examined, and it was determined that the three factor model was consistent with the data (*χ*^2^/df = 2.658, RMSEA = 0.062, SRMR = 0.031, CFI = 0.977, IFI = 0.977, GFI = 0.951). The factor loadings for the scale ranged from 0.622 to 0.932. The Cronbach’s Alpha coefficient for the scale as a whole was found to be 0.879. The findings reveal that the scale is a valid and reliable measurement tool within the scope of the current study.

Athlete Self-Efficacy Scale (ASES), developed by Koçak (2020) to measure athletes’ self-efficacy. The scale consists of 16 items and 4 subscales. The subscales of the 5-point Likert-type scale are defined as sport discipline efficacy (4 items), psychological efficacy (4 items), professional thought efficacy (4 items), and personality efficacy (4 items). As scores on the scale increase, it is interpreted that athletes’ self-efficacy levels also increase. The Cronbach Alpha internal consistency coefficient was determined to be 0.898 for the scale as a whole, while the subscales were calculated as 0.841, 0.756, 0.752, and 0.760, respectively (Koçak, 2020). The confirmatory factor analysis conducted to examine the factor structure of the self-efficacy scale revealed that the model had acceptable fit values (*χ*^2^/df = 2.733, RMSEA = 0.064, SRMR = 0.049, CFI = 0.931, IFI = 0.931, GFI = 0.928). The factor loadings of the items included in the scale were found to range between 0.348 and 0.807. The Cronbach Alpha coefficient for the scale as a whole was determined to be 0.882. These findings support the self-efficacy scale as a valid and reliable measurement tool within the scope of the research.

Psychological Skills Assessment Scale for Athletes (PSAS) developed by Smith et al. (1995) to assess seven different psychological skills in athletes. The scale was adapted into Turkish by Erhan et al. (2015). The scale consists of 28 items on a 4-point Likert scale. The scale consists of seven sub-dimensions: Ability to cope with difficulties, openness to learning, concentration, self-confidence and succeed motivation, goal setting and mental preparation, performing well under pressure, and getting rid of worries; each sub-dimension comprises four items. The Cronbach Alpha for the scale as a whole was calculated as 0.85. For the subscales, it was calculated between 0.50 and 0.71 (Erhan et al., 2015). As a result of the confirmatory factor analysis conducted to test the seven factor scale structure, it was determined that the factor loadings of some items were lower than expected. Specifically, Items 11, 23, and 25 were removed from the scale to improve model fit and ensure structural validity. After removing the three items with low factor loadings from the study, the analyses were redone, and the obtained fit indices (*χ*^2^/df = 2.222, RMSEA = 0.053, SRMR = 0.063, CFI = 0.898, IFI = 0.900, GFI = 0.911) showed that the model fit was acceptable. The factor load-ings on the scale ranged from 0.306 to 0.776, and the Cronbach’s Alpha coefficient for the scale as a whole was found to be 0.863. These findings demonstrate that the scale is a valid and reliable measurement tool within the scope of the current study.

Psychological Resilience Scale-Short (PRS-S) developed by Smith et al. (2008) to measure individuals’ levels of psychological resilience. The Turkish validity and reliability study was conducted by Doğan (2015). The scale is a single-dimensional instrument consisting of six items rated on a 5-point Likert-type scale. Higher scores on the scale are interpreted as indicating that individuals have greater psychological resilience. The internal consistency coefficient of the scale was calculated as 0.83 (Doğan, 2015). As a result of the confirmatory factor analysis of the six-item scale structure, it was determined that Item 5 had a low factor loading and was therefore removed to improve model fit and ensure structural validity. After removing the relevant item, the analysis showed that the model exhibited excellent fit values (*χ*^2^/df = 0.793, RMSEA = 0.000, SRMR = 0.011, CFI = 1.000, IFI = 1.000, GFI = 0.998). The factor loadings for the remaining items ranged from 0.387 to 0.800. The Cronbach’s Alpha coefficient calculated for the scale as a whole was 0.713, indicating that the scale provides an acceptable level of reliability in the current research context.

### 3.4 Procedure

The necessary permissions for the scales to be used in the study have been obtained. Subsequently, the study was approved as ethically sound by the Istanbul University-Cerrahpaşa Social and Human Sciences Research Ethics Committee in its decision numbered 491, dated August 05, 2025. Following ethical approval, the managers responsible for the academies of professional football clubs operating in Istanbul, as well as the families of the football players, were informed about the purpose and scope of the study. During the data collection process, the questionnaire was shared with participants via the WhatsApp groups of academy football players. In addition to the information provided by the managers, participants were also informed about the study through the explanations included in the questionnaire. Participation in the study was voluntary, and participants declared their willingness to participate by checking the consent box on the questionnaire.

### 3.5 Data analysis

SPSS 22 and AMOS 22 software were used to analyze the data in this study. Descriptive statistics were first applied, and it was determined that the data exhibited a normal distribution because the values ranged between −1.5 and +1.5. Furthermore, no multicollinearity issues were encountered in the data (Tabachnick and Fidell, 2013). The factor structures of the scales used were tested using Confirmatory Factor Analysis (CFA). The model constructed in the study was analyzed using Path Analysis within the Structural Equation Modeling (SEM) framework, and the Maximum Likelihood estimation method was used. In the mediation analyses, evaluations were based on Bootstrap confidence intervals. The model’s fit level was evaluated according to the criteria of a *χ*^2^/df value below 5, RMSEA and SRMR values below 0.08, and CFI, IFI, and GFI values above 0.90 (Kline, 2016). Furthermore, Harman’s (1976) one-factor test was employed to examine potential common method bias (CMB), thereby supporting the validity and reliability of the data.

## 4 Results

### 4.1 Common method bias test

To assess the potential presence of common method bias (CMB), Harman’s single factor test was conducted. All measurement items used in the study were subjected to an exploratory factor analysis using the Principal Axis Factoring method. The results showed that the first factor accounted for only 21.3% of the total variance. Since this value is well below the commonly accepted threshold of 40% (Tang and Wen, 2020), it can be concluded that common method bias is not a significant concern in this study. Therefore, the risk of systematic error due to the measurement method appears to be low.

Table 2 presents descriptive information regarding the scales. According to the findings, the perceived social support levels of academy football players (*M* = 61.23, SD = 14.65) are seen to be at a medium-high level. Self-efficacy (*M* = 4.28, SD = 0.47) and psychological skill scores (*M* = 3.25, SD = 0.36) were found to be above average, while psychological resilience levels (*M* = 22.09, SD = 3.94) were found to be at a moderate level. Skewness and kurtosis values were found to be within the ±1 range for all variables.

**Table 2:** Correlation results between variables.

Variable	*M*	SD	Skewness	Kurtosis	Range	1	2	3
1. Perceived social support	61.23	14.65	−0.464	−0.001	70.00	–		
2. Self-efficacy	4.28	0.47	−0.514	−0.244	2.19	0.285^**^	–	
3. Psychological skills	3.25	0.36	−0.396	−0.026	2.00	0.287^**^	0.722^**^	–
4. Psychological resilience	22.09	3.94	0.032	−0.008	24.00	0.208^**^	0.493^**^	0.560^**^

** *p* < 0.01.

When examining the correlation results between variables, it was determined that perceived social support showed positive and significant relationships with self-efficacy (*r* = 0.285, *p* < 0.01), psychological skills (*r* = 0.287, *p* < 0.01), and psychological resilience (*r* = 0.208, *p* < 0.01). A positive and significant relationship was found between self-efficacy and psychological skills (*r* = 0.722, *p* < 0.01) and a positive and significant relationship between psychological skills and psychological resilience (*r* = 0.493, *p* < 0.01). Furthermore, a positive and significant relationship was also found between psychological skills and psychological resilience (*r* = 0.560, *p* < 0.01).

**Figure 2:**
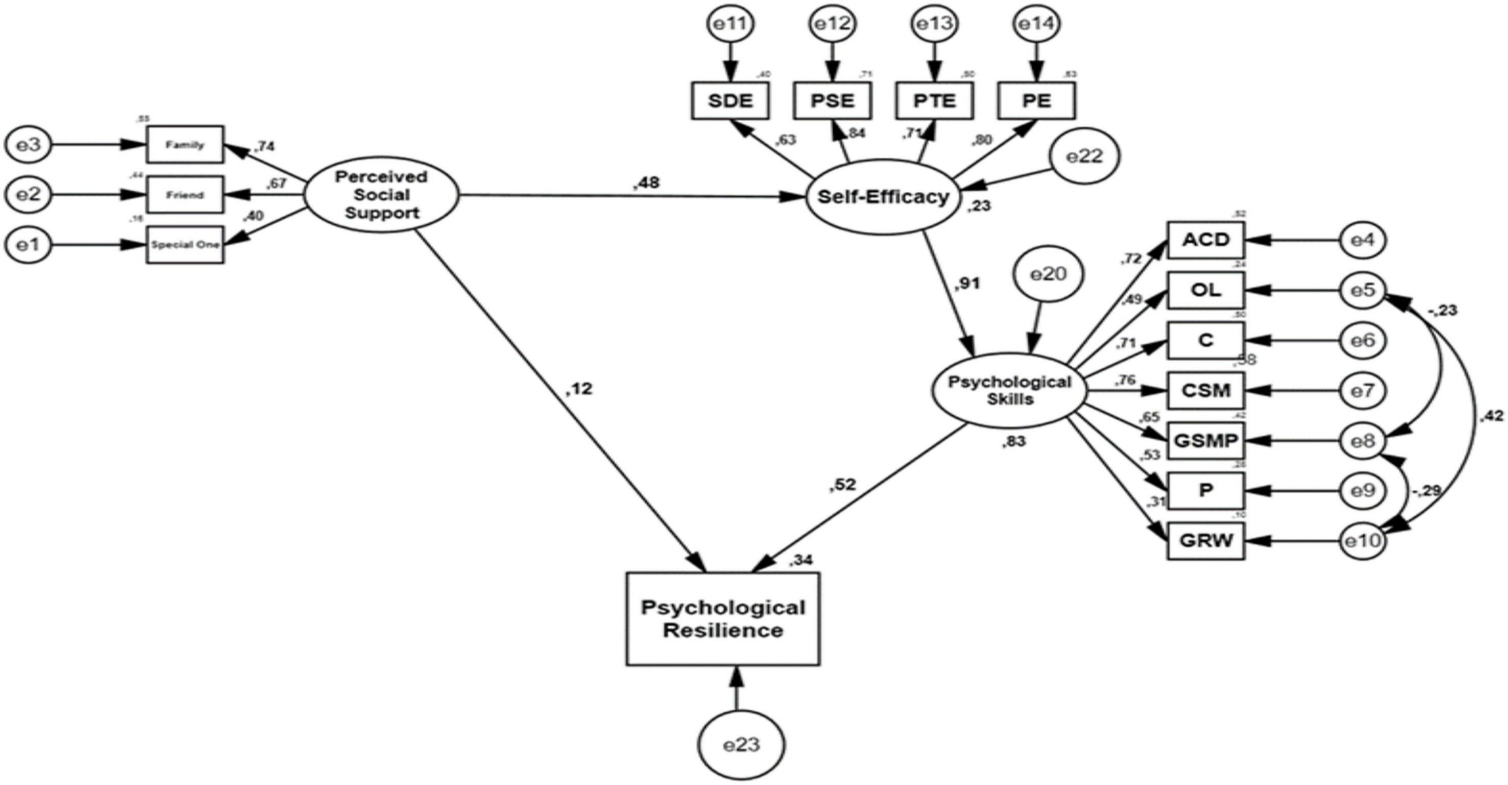
Structural model results.

The structural relationships between perceived social support, self-efficacy, psychological skills, and psychological resilience were tested using Structural Equation Modeling (SEM) (Figure 2). The fit indices obtained from the initial analysis (*χ*^2^/df = 4.891, RMSEA = 0.095, SRMR = 0.069, CFI = 0.864, IFI = 0.865, GFI = 0.872) were found to fall outside the acceptable reference range. After defining covariances between some error terms in the model, the analysis was repeated, and it was determined that the fit indices (*χ*^2^/df = 3.598, RMSEA = 0.078, SRMR = 0.057, CFI = 0.913, IFI = 0.913, GFI = 0.918) reached an acceptable level. These findings indicate that the proposed structural model is consistent with the data (Table 3).

**Table 3:** Model fit indices.

Fit index	Model 1	Model 2	Acceptable fit indices
*χ*^2^/df	4.891	3.598	<5.00
RMSEA	0.095	0.078	≤0.08
SRMR	0.069	0.057	≤0.08
CFI	0.864	0.913	≥0.90
IFI	0.865	0.913	≥0.90
GFI	0.872	0.918	≥0.90

According to the results of the Structural Equation Model in Table 4, the direct effect of perceived social support on self-efficacy is significant (*β* = 0.475; 95% CI [0.343, 0.589]). Self-efficacy significantly predicts psychological skills (*β* = 0.913; 95% CI [0.864, 0.952]), and psychological skills also have a significant effect on psychological resilience (*β* = 0.522; 95% CI [0.425, 0.629]). The direct effect of perceived social support on psychological resilience is not significant (*β* = 0.120; 95% CI [−0.015, 0.237]). In contrast, the indirect effect of perceived social support on psychological resilience through self-efficacy and psychological skills is significant (*β* = 0.226; 95% CI [0.158, 0.316]). This finding indicates that the effect of social support on psychological resilience occurs through indirect pathways and that full mediation is present. Furthermore, the total effect of social support on psychological resilience was found to be significant (*β* = 0.347; 95% CI [0.237, 0.455]).

**Table 4:** Chain mediation analysis predicting psychological resilience.

Direction of effect	Path relationship	Estimate	Boot SE	Bootstrap 95% CI	
				LLCI	ULCI
Direct effect	Perceived social support → self-efficacy	0.475	0.063	0.343	0.589
	Self-efficacy → psychological skills	0.913	0.022	0.864	0.952
	Psychological skills → psychological resilience	0.522	0.052	0.425	0.629
	Perceived social support → psychological resilience	0.120	0.065	−0.015	0.237
Indirect effect	Perceived social support → self-efficacy → psychological skills → psychological resilience	0.226	0.040	0.158	0.316
Total effect	Perceived social support → psychological resilience	0.347	0.056	0.237	0.455

## 5 Discussion

The primary aspiration of academy football players is to reach the professional level. However, throughout their careers, they may encounter various psychological challenges. Consequently, maintaining a high level of psychological resilience is crucial for their development and performance. Previous research has demonstrated that multiple factors can influence athletes’ psychological resilience, and perceived social support has been identified as one such potential factor. Nevertheless, it is suggested that perceived social support may not exert its effects in isolation; rather, these effects may be mediated by additional psychosocial variables. Against this backdrop, the present study aimed to examine the impact of perceived social support on psychological resilience among academy football players, as well as the sequential mediating roles of self-efficacy and psychological skills in this relationship.

In the constructed model, the initial examination of the relationship between perceived social support and psychological resilience revealed a statistically non-significant direct effect, leading to the rejection of H2. This key finding offers a critical theoretical nuance, as it diverges sharply from a significant body of the preceding literature that frequently reports a direct, positive association. For instance, studies on individual athletes such as Judokas (Chen et al., 2025; Şenel et al., 2025) and weightlifters (Shang and Yang, 2021), along with studies on specific populations like individuals with disabilities (Mira et al., 2023) and martial athletes (Moradi et al., 2023), have established clear direct links, often because support in those contexts directly enhances coping capacity or solitary performance belief. The non-significant direct effect in our study is likely a reflection of the unique structural characteristics and developmental context of professional football club academies. While previous research on college football players (Zhao et al., 2022; Liu et al., 2023) has shown a direct link, the demands on academy players differ significantly. The transition to the senior team is defined as ‘a complex career phase where players encounter simultaneous and multi-dimensional challenges to reach the professional level’. In this high-stakes environment, players must not only manage their personal development but also ‘a collaborative network of relationships that requires the effective management of a broad environmental structure encompassing internal and external stakeholders’ (European Club Association, 2024). This absence of a significant direct effect is a strong theoretical signal that the influence of perceived social support on resilience in this specific population is not direct, but instead channeled through personal resources. Psychological resilience in these young athletes is not merely an outcome of external support, but the product of an interaction between that support and their internal personal resources. Thus, the study confirms that variables like self-efficacy and psychological skills are likely to serve as essential mechanisms that convert perceived support into tangible gains in resilience, justifying their inclusion and examination within the proposed model. Future research could further investigate these mediating mechanisms and explore additional factors, such as coping strategies or motivational climate, that may moderate the relationship between perceived social support and psychological resilience in academy players.

Prior to examining the mediating roles of self-efficacy and psychological skills, the effect of perceived social support on the self-efficacy of academy football players was analyzed. The results indicated that perceived social support significantly and positively predicts self-efficacy, leading to the acceptance of H1. These findings align with previous research. For instance, Jiang et al. (2023) found that football players’ perceptions of coaching support positively influenced their self-efficacy. Similarly, other studies have reported that higher levels of perceived social support are associated with increased self-efficacy in athletes (Zou et al., 2023; Coussens et al., 2025). The positive impact of perceived social support on self-efficacy is particularly important for athletes’ performance development and mental toughness (Shi et al., 2025). In team sports such as football, social support not only provides emotional reassurance but also strengthens athletes’ self-confidence (Freeman and Rees, 2010). Academy football players face multiple uncertainties during their transition to professionalism, and support from family, coaches, and peers enhances self-efficacy, enabling athletes to perform with greater confidence in both training and competition. Consequently, social support serves as a key resource that fosters both psychological resilience and motivation. Furthermore, the findings highlight the central role of self-efficacy in the development of athletes’ psychological skills. Athletes with higher self-efficacy are more adept at psychological skills such as stress coping, and maintaining motivation (Guo et al., 2019; Tokarska and Rogowska, 2025). Elevated self-efficacy reinforces fundamental psychological competencies, including recovery after mistakes, sustaining self-confidence, and goal commitment (Munroe-Chandler et al., 2008; Çetin et al., 2021; Yılmaz et al., 2024). Moreover, high levels of self-efficacy have been shown to enhance athletes’ proficiency in psychological skills such as stress management, concentration, and emotional regulation (Bandura, 1997; Lowther et al., 2002; Feltz et al., 2008), further supporting the conceptual link between self-efficacy and psychological skill development. These results are further supported by evidence from other sports contexts. For example, Volgemute et al. (2024) demonstrated that improvements in self-efficacy among youth alpine skiers were accompanied by significant enhancements in imagery abilities, which in turn improved task performance, highlighting the interdependent nature of psychological skills and self-efficacy. Similarly, Hasnan et al. (2025) found strong positive correlations between self-efficacy and life skills in young athletes, indicating that higher self-efficacy enhances athletes’ ability to cope with challenges, regulate performance demands, and maintain persistence, abilities closely related to psychological skills. Furthermore, Sivrikaya (2019), in a study with football players, reported that self-efficacy significantly contributes to skill acquisition and performance improvement, reinforcing the notion that self-efficacy functions as a foundational psychological resource that strengthens both task-specific and broader psychological competencies. Within this conceptual framework, the sequential mediating roles of self-efficacy and psychological skills in the relationship between perceived social support and psychological resilience were examined. The results supported H3, indicating that the effects of perceived social support on psychological resilience are indirect and occur through self-efficacy and the psychological skills it fosters. This highlights that while social support may not exert a significant direct effect on resilience in academy players, it indirectly contributes by enhancing personal resources such as self-efficacy, which then facilitate the development of psychological skills essential for resilience. As noted by Sarkar and Fletcher (2014), athletes’ psychological resilience is influenced not only by personal characteristics but also by social support and environmental factors. Overall, these findings suggest that self-efficacy and psychological skills play a central mediating role in explaining how perceived social support influences psychological resilience. The observation that social support contributes indirectly via self-efficacy rather than directly to resilience provides valuable insights into the mechanisms underlying athletes’ developmental processes and underscores the importance of considering both environmental and personal resources in high-performance contexts.

## 6 Conclusion

The present study examined the underlying factors associated with psychological resilience among football players competing in the academies of professional football clubs. The findings make an original contribution to the field by demonstrating that perceived social support does not exert a direct effect on psychological resilience; rather, its influence operates entirely through self-efficacy and psychological skills. This result indicates that the development of resilience is not solely contingent upon external environmental factors, but is also critically dependent on athletes’ capacity to internalize these environmental resources and to cultivate personal assets such as self-efficacy beliefs and psychological skills.

### 6.1 Practical implications

The findings provide important practical implications for athlete development strategies involving coaches, club management, and sport psychologists. Rather than adopting approaches solely focused on performance enhancement, coaches and club administrators are encouraged to implement development-oriented strategies aimed at strengthening athletes’ self-efficacy beliefs and psychological skills, such as stress management, concentration, and motivation. Coaches should place emphasis not only on competitive outcomes but also on athletes’ effort, developmental processes, and individual progress, and should provide regular feedback that makes tangible improvements visible and reinforces athletes’ self-efficacy beliefs. Furthermore, maintaining family support and enhancing the quality of social support resources available within the academy environment may play a complementary role in fostering athletes’ long-term psychological resilience.

### 6.2 Limitations

The present study has several limitations that should be acknowledged. First, the use of self-report measures may introduce the risk of common method bias. Future research may address this limitation by incorporating additional data sources, such as objective performance indicators or coach evaluations. Second, the sample was restricted to players competing in the academies of professional football clubs, which limits the generalizability of the findings. Future studies may enhance external validity by including athletes from different sports disciplines and varying developmental levels. Finally, the cross-sectional design of the study precludes definitive conclusions regarding causal relationships among the variables. Longitudinal research designs would allow for a more comprehensive examination of changes over time in the contributions of social support, self-efficacy, and psychological skills to psychological resilience.

## Data Availability

The data that support the findings of this study are available from the corresponding author, AS, upon reasonable request.
